# Associations Between Experienced and Internalized HIV Stigma, Adversarial Growth, and Health Outcomes in a Nationwide Sample of People Aging with HIV in Germany

**DOI:** 10.1007/s10461-020-03061-3

**Published:** 2020-10-14

**Authors:** Jochen Drewes, Phil C. Langer, Jennifer Ebert, Dieter Kleiber, Burkhard Gusy

**Affiliations:** 1 Public Health: Prevention and Psychosocial Health Research, Freie Universität Berlin, Habelschwerdter Allee 45, 14195 Berlin, Germany; 2 International Psychoanalytic University, Stromstr. 3b, 10555 Berlin, Germany

**Keywords:** HIV stigma, Posttraumatic growth, Mental health, Self-rated health, Mediation

## Abstract

HIV-related stigmatization and adversarial growth are known to influence health outcomes in people living with HIV. But not much is known how these psychosocial factors are related to each other and how they interact to influence health outcomes. We tested whether the effect of experienced and internalized stigma on mental health and self-rated health is mediated by adversarial growth, and whether each of these factors is uniquely associated with health outcomes. In our sample of 839 people aging with HIV in Germany based on a cross-sectional study design we did not find an indirect effect of experienced HIV stigma on health outcomes and a very small indirect effect of internalized HIV stigma. All variables were significant predictors of health outcomes in multiple regression analyses.

## Resumen

Se sabe que el estigma y el crecimiento postraumático asociados al VIH influyen en los indicadores del estado de salud de las personas que viven con el virus. Sin embargo, se desconoce cómo estos factores psicosociales se relacionan entre sí, y cómo interactúan sobre los indicadores del estado de salud. En este estudio comprobamos si los efectos de la experiencia e internalización del estigma sobre la salud mental y la evaluación subjetiva del estado salud están mediados por el crecimiento postraumático, así como el impacto único de cada uno de estos factores sobre los indicadores de salud. Usando un diseño de estudio transversal en una muestra de 839 personas que viven con VIH en Alemania, no encontramos un efecto indirecto del estigma experimentado en los indicadores de salud, pero sí, un efecto indirecto muy pequeño del estigma internalizado. Todas las variables se mostraron predictores significativos de los resultados de salud en un análisis de regresión múltiple.

## Introduction

Even in the era of antiretroviral treatment (ART), living with an HIV infection is characterized by impaired mental and physical health, and negative and positive psychosocial factors are known to influence health outcomes among people living with HIV (PLWH) [1, 2]. HIV-related stigmatization and growth processes after a ‘traumatic’ HIV diagnosis are two of these factors. Not much is known about how these two factors are related to each other and whether they interact to influence health outcomes.

### HIV-related Stigma and Health Outcomes

Since it was first described HIV infection is a highly stigmatized condition [3] and PLWH are exposed to more stigmatization than people living with other diseases [4]. This is true even since the advent of ART normalized HIV infection to a manageable chronic disease, though there is evidence that ART has the potential to diminish the stigmatization of PLWH [5]. A widely agreed upon definition of the stigma concept in general or HIV stigma in particular does not exist [6], however, most authors define stigma via its manifestations or mechanisms (e.g. [7]). While stigma can manifest on the level of the stigmatizer, the stigmatized and the society, we will focus here on the perspective of the target of stigmatization. In their HIV Stigma Framework, Earnshaw and Chaudoir [8] differentiated between three manifestations or mechanism of stigma that affect the stigmatized: 1) experienced stigma (ES; the actual experience of discrimination), 2) anticipated stigma (AS; the expectation of experiencing discrimination in the future), and 3) internalized stigma (IS; the endorsement of stigmatizing beliefs and feelings about oneself like feelings of guilt, shame and self-blaming). The different mechanisms of HIV stigma are neither conceptually nor statistically independent from each other. Indeed, the authors reported significant correlations between AS and ES and between IS and AS but could not detect a significant relationship between IS and ES [9]. However, an innovative study, using an experience sample approach could demonstrate that the experience of discriminative acts predicted increases in IS among PLWH [10].

The detrimental consequences of HIV-related stigmatization for the health of PLWH are well documented. Extensive research shows that stigma is related to distress, depression and anxiety, overall quality of life, physical health, social support, adherence to antiretroviral therapy and other health behaviors, as well as access to and usage of health services in PLWH (for a review see [11]). While associations with all health indicators are of substantial dimensions, relationships with mental health are generally stronger than with physical health outcomes. Meta-analyses calculated effect sizes ranging from 0.30 to 0.41 between stigma and depression and/or anxiety symptoms while effect sizes for physical health ranged from − 0.19 to − 0.32 [11, 12]. Unfortunately, these meta-analyses were not able to differentiate between stigma manifestations, as most instruments to assess stigma in PLWH are not designed to assess all three manifestations separately. Research on stigma that employs Earnshaw and Chaudoir’s HIV Stigma Framework is still in an early stage of development. However, a study by Earnshaw and colleagues demonstrated that all three stigma manifestations are independently related to various health outcomes. IS showed the biggest association with mental health aspects while ES was strongest related to physical health outcomes [9]

### Adversarial Growth in People Living with HIV and Health Outcomes

Experiencing an adverse, potentially traumatic event like the diagnosis of an HIV infection is not necessarily restricted to negative psychological or health outcomes. In the contrary, there is evidence that people experience positive changes in their life while dealing with an adverse event. These changes are referred to in the literature as growth processes and come under many interchangeable terms like e.g. adversarial growth (AG), posttraumatic growth (PTG), or benefit finding. For this paper, we will use the broader term “adversarial growth” first proposed by Linley and Joseph [13]. These authors define AG as the result of “struggling with adversity”, AG in this respect relates to any changes following an adverse event, which “propel the individual to a higher level of functioning than that which existed prior to the event” [13]. This way we avoid the connotations that come with the clinical term ‘posttraumatic’ and stress the fact that the growth processes we are researching are not depending on the existence of ‘trauma’ but on a variety of adverse events.

Different theoretical models were proposed to explain the AG phenomenon, however, according to Sawyer et al. most models have in common that “the experience of a highly stressful or traumatic event violates an individual’s basic beliefs about the self and the world and that some type of meaning making or cognitive processing to rebuild these beliefs and goals occur, resulting in perceptions that one has grown through the process” (p. 437) [14]. Dimensions on which growth is occurring also vary across models, but most models and instruments embrace these three dimensions: 1) changes in personal/inner strength, 2) changes in life philosophy or appreciation of life and 3) changes in relationships with others [15].

Originally, theories on AG were not concerned with potential health outcomes of the growth process. However, some studies analyzed these relationships between AG and mental and physical health. In general, the research results regarding this question are inconclusive [16] and there are considerably less empirical studies on this research question compared to studies on HIV stigma and health outcomes. The best empirical evidence exists for an inverse relationship between adversarial growth and depression in PLWH with the majority of studies showing significant relationships between both variables [17], with small to moderate effect sizes [18]. It is difficult to identify similar trends in respect to anxiety symptoms or other health outcomes because of the small number of studies researching these relationships [19]. Existing studies show a significant inverse relationship between AG and anxiety [19, 20] and a significant positive relationship with the physical functioning domain of quality of life [21], while another study reports a non-significant relationship with self-rated health [22]. In a sample of elderly PLWH AG was associated with better cognitive aging [23].

### The Relationship Between Stigma and Adversarial Growth

Research on the relationships between stigma manifestations and AG is just recently emerging. In models of AG stigma is not considered as a variable as these models do not focus on growth processes elicited by health-related conditions like HIV/AIDS.

In the five published empirical studies that report relationships between HIV-related stigma and AG, stigma is in each case conceptualized as a predictor of AG. Each of these studies hypothesize a negative effect of stigma on AG based on the assumption that stigma is hindering growth processes. In the theoretically most elaborate approach, Kamen et al. [24] propose a direct and an indirect way of how stigma has a negative impact on growth processes based on Tedeschi and Calhoun’s seminal model of PTG [15]. The indirect effect hypothesizes that stigma hinders HIV status disclosure, which is essential for receiving social support, which is again essential for growth to occur. In a direct way stigma may “decrease PTG by hampering individuals’ ability to appropriately express emotion, develop an enhanced understanding of their trauma, and begin the process of meaning-making” (p. 127) [24]. Other conceptualizations of the relationship between stigma and AG are also possible. Garrido–Hernansaiz and colleagues referred to theoretical assumptions about the role of distress in the growth process explaining their surprising positive correlation between stigma and AG [25]. While, in the times of a normalization of HIV the infection itself may not be able to generate stress levels that are sufficiently high to initiate growth processes, the existence of stigma could contribute to a stress level that is sufficient for growth processes to occur.

The results of these studies are inconclusive concerning the relationship between stigma and AG in PLWH. Four of these studies showed small to medium sized negative correlations between measures of HIV-related stigma and AG. While in two studies bivariate correlations between both variables were statistically significant (r > 0.20), the relationship was non-significant when controlled for other variables like resilience or social support [22, 26]. Kamen et al. reported smaller correlations between adversarial growth and HIV stigma and disclosure behavior, but after adding social support to the regression term only disclosure behavior stayed a significant predictor of adversarial growth [24]. Dibb, however, did not find a significant bivariate relationship between AG and HIV stigma but a surprisingly high bivariate negative association between AG and disclosure regret (r = − 0.46) [27]. Contrarily, Garrido–Hernansaiz and colleagues reported positive direct and indirect relationships between IS and AG using a structural equation modelling approach [25].

### Current Study

The current study focusses on the associations between the two HIV stigma manifestations ES and IS, and AG and health outcomes in people living with HIV. Based on previous research on the associations between HIV stigma and AG we will first analyze if AG works as a mediator between these stigma manifestations and two health outcomes, mental health and self-rated health. This involves the testing of four hypotheses: H1) AG (partially) mediates the effect of ES on mental health. H2) AG (partially) mediates the effect of ES on self-rated health. H3) AG (partially) mediates the effect of IS on mental health. H4) AG (partially) mediates the effect of IS on self-rated health.

In a second step we will analyze the unique independent effects of ES, IS and AG on both health outcomes. This involves the testing of six hypotheses: H5) ES is an independent predictor of mental health. H6) ES is an independent predictor of self-rated health. H7) IS is an independent predictor of mental health. H8) IS is an independent predictor of self-rated health. H9) AG is an independent predictor of mental health. H10) AG is an independent predictor of self-rated health.

## Methods

### Study Design and Participants

The study *50plushiv* was a cross-sectional, exploratory study with a quantitative and a qualitative study arm to describe the health, living conditions and needs of people aging with HIV and AIDS in Germany conducted in 2013–2014. In this paper, we will report results from the quantitative study arm. Eligible participants were all people with a self-reported diagnosis of HIV, 50 years old or older and living in Germany. The questionnaire was provided as an online questionnaire and as a paper pencil questionnaire. Several online and offline strategies were used to reach potential participants.

A total of 907 people who met the eligibility criteria completed either the paper (n = 499) or the online (n = 408) questionnaire. We eliminated all participants with missing data on one of the study variables we used for the mediation and regression analyses (n = 68). Our final analysis sample consisted of n = 839 participants.

### Measures

#### Sociodemographic Variables

Sociodemographic variables were used for descriptive purposes and as covariates in the multiple regression analyses. These variables comprise age, gender, sexual orientation, HIV transmission group, duration of HIV infection and education. Age and duration of HIV infection were used as metric variables in the regression analyses, gender, education, sexual orientation and HIV transmission group were dummy coded for regression analyses.

#### Experienced HIV Stigma

We assessed ES with a self-constructed index that we used in previous unpublished research because we were not convinced by the wording and content of existing measures (see Online Appendix for items). Participants were asked to rate how often they experienced various forms of stigmatization and discrimination because of their HIV infection on a 5-point Likert scale (never–very often). Items were operationalizing blaming and shaming (e.g. “Friends are blaming me for my HIV infection”), distancing (“Potential (sex) partners are rejecting me because of my HIV infection”) and structural stigma (“I was denied medical aid or was treated unfair at a physician’s practice, hospital or other health institution”). We assigned scores from 0 (never) to 4 (very often) and added scores to a single sum score with a possible range from 0 to 28. Our index of experienced stigma with 7 items had an acceptable internal consistency with Cronbach’s alpha = 0.78.

#### Internalized HIV Stigma

IS was assessed using the negative self-image scale of the HIV Stigma Scale [28] in the German translation [29]. The HIV Stigma Scale is the most widely used scale to measure HIV-related stigma from the perspective of the stigmatized [12]. For this study, we used 6 out of 7 items from the original scale; one item was dropped due to human error in the construction of the questionnaire. Responses were recorded on a 4-point Likert scale (strongly agree–strongly disagree). Internal consistency for our scale of 6 items was good: Cronbach’s alpha = 0.89.

#### Adversarial Growth

We used an abbreviated version of the Silver Lining Questionnaire (SLQ) [30, 31] to measure adversarial growth. The SLQ-38 is a 38-item questionnaire constructed to measure growth processes following the diagnosis of a chronic illness. While the authors Sodergreen and Hyland claim that the SLQ-38 is unidimensional, Bride et al. [32] revisited the factor structure of the instrument and presented an abbreviated instrument with 24 items loading on five factors. As space limitations in our survey did not allow the integration of a 24-item scale the instrument was even more abbreviated by selecting only the two highest loading items from four of the five factors proposed by Bride et al. a) improved personal relationships, b) greater appreciation for life, c) personal inner strength and d) changes in life philosophy. In this study a 4-point Likert scale (strongly agree – strongly disagree) was used as suggested by Sodergreen & Hyland of this paper to avoid the middle “not sure” option. We also followed Bride et al.’s suggestion to use continuous scoring instead of Sodergreen and Hyland’s dichotomization of the response options. Items were translated by the authors using standard procedures. Preliminary factor analyses showed that all 8 items loaded highly (> 0.70) on one shared factor (principal component analysis: Eigenvalue = 4.8, explains 59.8% of variance). The 8-item scale showed excellent internal consistency with Cronbach’s alpha = 0.90.

#### Mental Health

The PHQ-4 scale, an ultra-brief screening instrument for Major Depression and other forms of depression as well as for Generalized Anxiety Disorder and other anxiety and panic disorders [33], was used to measure mental health. The PHQ-4 items are asking for the frequency of symptoms experienced in the last 14 days. Responses are scored from 0 (“not at all”) to 3 (“nearly every day”) and added to obtain a simple sum score ranging from 0 to 12. Scores are categorized in four groups for descriptive purposes: normal (0–2), mild (3–5), moderate (6–8), severe (9–12). While we use these categorizations for descriptive purposes, the sum score was used as an “overall measure of symptom burden, as well as impairment and disability” [33] in our mediation and regression analyses. Cronbach’s alpha for the PHQ-4 in our sample was excellent: alpha = 0.90.

#### General Health Status

We assessed general health status with the item “In general, how would you rate your health?” Answers were recorded on a 5-point Likert scale (excellent–poor). This measurement of self-rated health is widely used in epidemiological studies, is part of various quality of life instruments and proved to be predictive of mortality in diverse populations [34].

### Statistical Analyses

All statistical analyses were conducted with IBM SPSS Statistics 25. We used the PROCESS version 3.3 procedure for SPSS by Hayes [35] to perform simple mediation analyses (model 4) to detect mediation effects between stigma manifestations, adversarial growth and health outcomes. To quantify independent effects of stigma manifestations and adversarial growth on health outcomes a multiple regression analysis was conducted for each health outcome controlling for age, education (dummy-coded: 10 years and less vs. more than 10 years), gender (dummy-coded: male vs female), sexual orientation (dummy-coded: heterosexual vs homosexual) and duration of HIV infection.

## Results

### Sample Characteristics and Intercorrelations Between Study Variables

Sample characteristics and descriptive statistics for the study variables can be found in Table 1. All study variables correlate significantly with each other in bivariate correlation analyses except experienced HIV stigma and adversarial growth (r = 0.032; p > 0.05). The highest correlations are found between mental health and self-rated health (r = 0.514; p < 0.001) and between internalized HIV stigma and mental health (r = 0.480; p < 0.001) (see Table 2).

**Table 1:** Descriptive statistics for sociodemographic and study variables (n = 839)

	%	Mean (Standard deviation)	Range
Age		56.9 (6.3)	50–83
50–59 years	73.5		
60–69 years	20.2		
70 years and older	6.3		
Gender			
Male	88.2		
Female	11.8		
Sexual orientation			
Heterosexual men	7.9		
Homo-/bisexual men	80.3		
Heterosexual women	10.7		
Homo-/bisexual women	1.1		
Education		12.4 (4.0)	3.3–21
10 years or less	55.3		
more than 10 years	44.7		
HIV transmission group			
Homosexual	76.8		
Heterosexual	13.8		
i.v. drug use	3.3		
Blood products	3.4		
Other/don’t know	2.7		
Duration of HIV-infection		16.7 (8.6)	1–32
1–5 years	11.7		
6–10 years	17.4		
11–20 years	34.0		
21 years and longer	37.0		
Experienced HIV stigma		3.5 (3.9)	0–24
Internalized HIV stigma		1.5 (0.7)	1–4
Adversarial growth		2.3 (0.8)	1–4
Mental health		3.4 (3.0)	0–12
Normal	44.2		
Mild	36.5		
Moderate	12.0		
Severe	7.3		
General health status		3.0 (.9)	1–5
Excellent	4.9		
Very good	21.3		
Good	50.0		
Fair	20.1		
Poor	3.7

**Table 2:** Intercorrelation matrix of study variables (n = 839)

	1	2	3	4
1 Experienced HIV stigma				
2 Internalized HIV stigma	**0.341**			
3 Adversarial growth	0.032	− **0.216**		
4 Mental health	**0.346**	**0.480**	− **0.179**	
5 Self-rated health	**0.287**	**0.289**	− **0.174**	**0.514**

Correlations in bold are significant at the 0.001 level

### Mediation Analyses

**Fig. 1:**
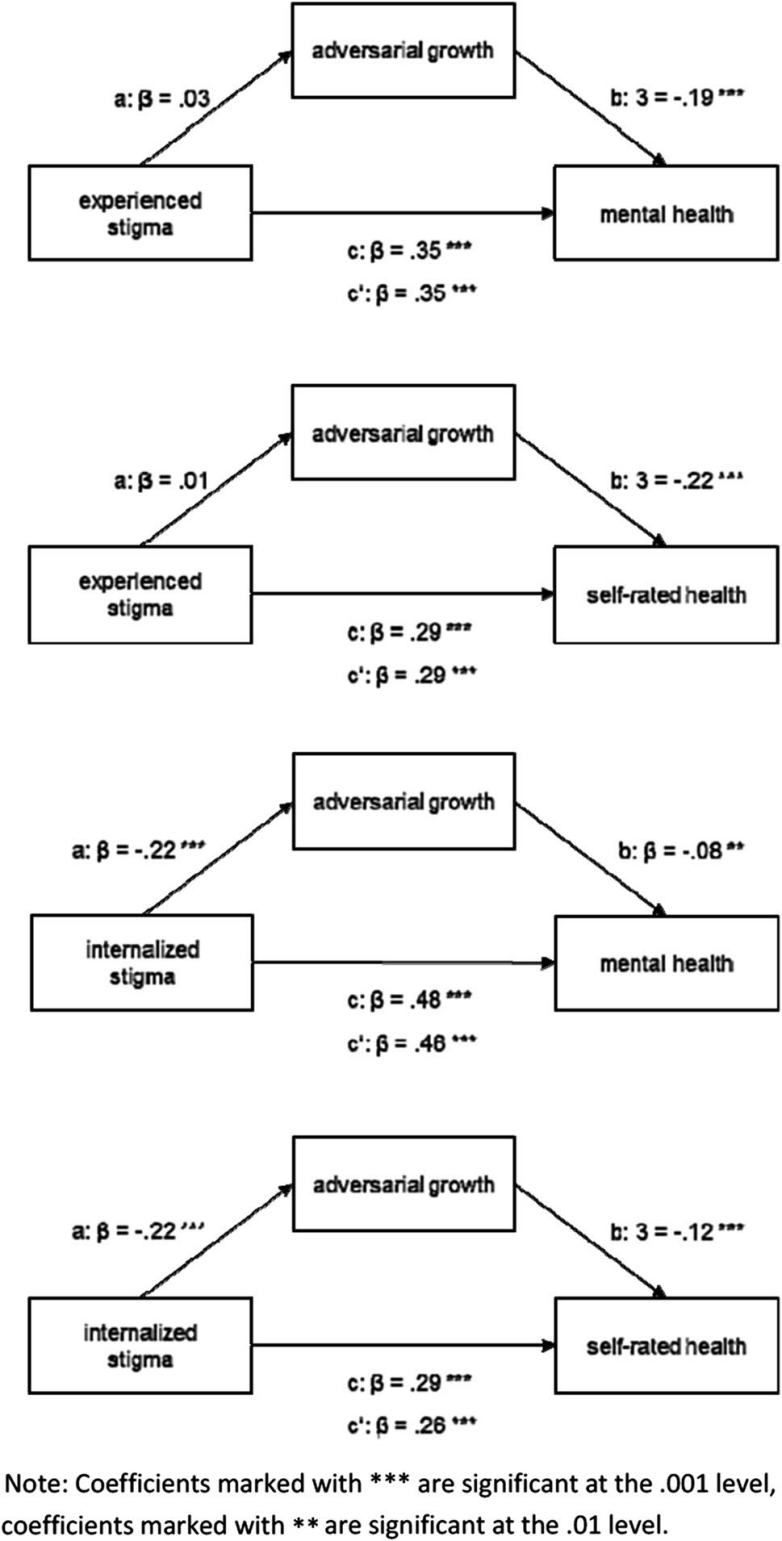
Mediation models with standardized coefficients for effects (n = 839). Note: Coefficients marked with ***are significant at the .001 level, coefficients marked with **are significant at the 0.01 level

Results of the mediation analyses can be found in Table 3 and in Fig. 1. ES was not a significant predictor of AG in the mediation analyses on mental health (B = 0.006; p = 0.373) and self-rated health (B = 0.006; p = 0.393) and both analyses did not show a significant indirect effect (for the mental health model: effect = − 0.005; 95% CI [− 0.015; 0.005] and for self-rated health: effect = − 0.001; 95% CI [− 0.005; 0.002]. Each of the mediation models regarding IS showed a significant indirect effect, for mental health the effect is 0.075 [95% CI (0.019; 0.143)] and for self-rated health the effect is 0.032 [95% CI (0.013; 0.054)].

**Table 3:** Results of the mediation analyses (n = 839)

	Predictor	Outcome	B (SE)	t	p
c	ES	MH	0.263 (0.026)	10.074	< 0.001
a	ES	AG	0.006 (0.007)	0.891	0.373
b	ES|AG	MH	− 0.768 (0.131)	− 5.887	< 0.001
c’	AG|ES	MH	0.268 (0.025)	10.695	< 0.001
c	ES	SRH	0.064 (0.007)	8.634	< 0.001
a	ES	AG	0.006 (0.007)	0.856	0.393
b	ES|AG	SRH	− 0.215 (0.040)	− 5.438	< 0.001
c’	AG|ES	SRH	0.065 (0.007)	8.933	< 0.001
c	IS	MH	2.101 (0.152)	13.831	< 0.001
a	IS	AG	− 0.234 (0.036)	− 6.567	< 0.001
b	IS|AG	MH	− 0.320 (0.122)	− 2.626	< 0.01
c’	AG|IS	MH	2.026 (0.153)	13.230	< 0.001
c	IS	SRH	0.371 (0.044)	8.372	< 0.001
a	IS	AG	− 0.235 (0.036)	− 6.609	< 0.001
b	IS|AG	SRH	− 0.137 (0.040)	− 3.393	< 0.01
c’	AG|IS	SRH	0.338 (0.045)	7.554	< 0.001

*c* total effect of predictor on outcome, *a* effect of predictor on mediator, *b* effect of mediator on outcome controlling for predictor, *c’* effect of predictor on outcome controlling for mediator (direct effect of predictor). *ES* experienced stigma, *AG* adversarial growth, *MH* mental health, *SRH* self-rated health, *IS* internalized stigma

### Regression Analyses

Table 4 shows the results of the regression analyses. After controlling for age, gender, education, sexual orientation, and duration of HIV infection, ES, IS and AG were significant predictors of mental health and self-rated health.

**Table 4:** Stigma manifestations and adversarial growth as predictors of mental health and self-rated health: multiple regression models (n = 839)

	Mental health				Self-rated health			
	β	B	Std F	T	β	B	Std F	T
Experienced HIV stigma	**0.192**	0.146	0.024	6.058	**0.215**	0.048	0.008	6.219
Internalized HIV stigma	**0.390**	1.709	0.146	11.742	**0.192**	0.245	0.046	5.280
Adversarial growth	− **0.106**	− 0.427	0.121	− 3.527	− **0.150**	− 0.177	0.039	− 4.577

Coefficients in bold are significant at the 0.001 level. All models are adjusted for age, gender, education, sexual orientation, and time since HIV diagnosis (entered as block 1)

## Discussion

The goal of this study was to further our understanding of the associations between stigma manifestations, adversarial growth and health outcomes. We tested ten hypotheses using mediation analyses and multiple regression analyses. Based on our results we have to reject hypotheses H1 and H2, as ES is not a significant predictor of AG and thus AG cannot mediate the effect of ES. Hypotheses H3 and H4 are confirmed by our data. IS has a significant indirect effect on mental health and self-rated health through AG in our mediation analyses, but both indirect effects are very small. Hypotheses H5 to H10 concerning the unique effects of the two stigma manifestations and AG on health outcomes are all confirmed by our data. ES, IS and AG each were significantly related to mental health and self-rated health in multiple regression analyses after controlling for age, education, gender, sexual orientation and duration of HIV infection.

### Theoretical Implications

While previous studies already looked into the relationship between HIV stigma and AG, we were the first to analyze the associations between AG and two of the three stigma mechanisms proposed by Earnshaw and Chaudoir [8, 9] and test mediations models to understand whether AG (partially) mediates the effect of stigma on health outcomes. In our sample IS and ES were unexpectedly in a different way related to AG. While IS was significantly correlated with AG and the magnitude of the association was equivalent to that found in other studies [22, 26], ES was in correlation and mediation analyses positively but statistically not significantly associated with AG. We could not identify any previous study that reported a correlation between a measure of ES and AG but one study reported a positive association between a measure of HIV-related stigma and AG [25]. In this study the measure of IS was associated positively associated with AG using structural equation modelling in a longitudinal research design. The authors of this study argue that stigma functions as an (additional) stressor and elevated stress levels are a prerequisite for growth processes (see introduction). While in this study an instrument measuring IS and not ES was used and this result in regard to IS stands in contrast to the results of any other study using a similar measure of IS, including our study, the notion that stigma-induced stress can foster growth processes should not be easily dismissed. The experience of stigma and discrimination indeed leads to stress reactions for the stigmatized individual and thus can be understood as a stressor [36], and IS, too, is associated with increased stress levels for the individual [37]. However, while both ES and IS work as a stressor for the stigmatized individual, we propose that only the stress associated with ES is able to instigate growth processes. IS is defined by feelings and cognitions like shame, self-blame, and worthlessness [8, 9], and these feelings and cognitions hinder growth processes: “PLWH who feel shameful about their HIV status may struggle to develop or maintain positive cognitions about their HIV“ (9, p. 1786). In Joseph et al.’s *affective-cognitive processing model of post-traumatic growth* two appraisal processes characterize the intrusion phase: a) ruminative brooding and b) reflective pondering [38]. While reflective pondering is required for the reappraisal of the adverse event and to eventually leave the intrusion phase, ruminative brooding can reinforce ‘negative emotional states such as guilt, shame or anger’ and ‘prolongs the intrusion phase’ [38, p. 322]. While both IS and ES was associated with rumination in previous studies [39, 40] and it is likely that both intensify ruminative brooding in the intrusion phase, ES leaves space for reflective pondering, but IS does not.

The fact that our measure of ES was not significantly related to AG can be explained taking the age of our participants and the lack of a time frame for our measure of ES into account. Our participants are aging with HIV and are at least 50 years or older, over 70% were diagnosed with HIV more than 10 years ago. Previous research showed that levels of stigma decrease with age and time since diagnoses [41, 42], while a similar effect for AG does not exist [17]. Our instrument to measure ES did not specify a time frame but asked for the frequency with which stigmatizing episodes were experienced by the participant at the moment or in the past. It is possible that stigmatizing events were either underreported due to the open time frame, or that these events took place so long ago that they did not have any effect on current AG levels. Moreover, it is possible that these events took place in the IS pre-growth phase, thus increasing IS levels instead of fostering growth. Our cross-sectional study design is not able to shed light on these processes and interactions of stigma and AG over the course of HIV infection.

While IS was strongly correlated with AG, the small mediation effect we detected on either health outcome does not support the hypothesis that AG is buffering the adverse effect of HIV-related stigma on mental health and self-rated health. However all stigma manifestations and AG had a unique impact on health outcomes. Stigma manifestations had higher impacts on health outcomes compared to AG, and AG had a higher impact on self-rated health than on mental health. We were able to replicate Chaudoir et al.’s findings that IS is stronger related to mental health than ES and, however less distinctive, ES is stronger related to physical health outcomes. The less distinctiveness of the latter finding might be due to the measure of physical health we chose. Self-rated health is not an exclusive measure of physical health, and studies show that the presence of depression and depressive symptoms has a substantial impact on the assessment [43]. This might explain the high correlation found between self-rated health and depressive and anxious symptoms in our sample.

Limitations

Our study has several limitations. We used data from a cross-sectional study, which means we cannot infer causality. Furthermore, mediation analyses require longitudinal data to obtain valid results, our results are thus to be interpreted with caution and seen as exploratory findings which need confirmation from studies with longitudinal designs. The instruments we used to assess HIV stigma and AG show some limitations too. While all previous studies on the associations between HIV stigma and AG used the Posttraumatic Growth Inventory [44], we chose the Silver Lining Questionnaire because this instrument was designed specifically to assess disease-related growth processes. Furthermore, we shortened the original SLQ from 36 to 8 items due to space restrictions. While the face validity of our instrument is still high, we do not know whether the shortened instrument was able to catch all aspects of adversarial growth that were assessed in the original version of the instrument or in similar instruments. We further found that the selected eight items loaded highly on one shared factor; a result already reported by the authors of the original 36 item scale [30]. Our results do not support the factor solution proposed by Bride et al. [32], though we chose only the two items that loaded highest on four of their five extracted factors—a result that could be also attributed to the much smaller number of items we used for our factor analysis. The instrument we used to measure ES was an ad hoc scale. While this measure shows a high face validity, we do not know whether it captures all relevant aspects of ES.

### Practical Implications

Our results show that ES, IS and AG have each a unique effect on health outcomes of PAWH. Interventions that focus on decreasing levels of ES or IS in PLWH can be effective in improving health outcomes in this population. The same applies for interventions that foster growth processes. While interventions that are specifically designed for this aim are still rare and lack rigorous evaluation of their effectiveness, a recent meta-analysis could demonstrate that existing psychosocial interventions can increase levels of AG [45]. Our study also implies that fostering growth processes to buffer the negative impact of internalized stigma on health outcomes may not be an effective strategy, but interventions to reduce levels of IS, can help to enable growth processes in PLWH.

### Implications for Future Research

Research on stigma generally lacks theoretical conceptualizations to inform research designs and evidence-based interventions [46]. Compared to the literature on AG, which is characterized by several theoretical approaches with elaborate conceptualizations of the growth process, and its prerequisites and consequences, there are only a few similar theoretical elaborations describing, for example, the process of the internalization of stigma or the mechanisms by which stigma leads to adverse health outcomes [39]. To improve our understanding of the trajectories and interactions between HIV stigma manifestations and AG methodologically sound longitudinal research designs are needed that are guided by theoretical frameworks. Ideally, these studies would start with patients who are recently diagnosed with HIV and monitor stigma manifestations, AG and health outcomes over an extended period of several years.

## Appendix

Experienced HIV stigma index

How often have you experienced the following in the past?

(very often–often–sometimes–rarely–never)

Friends and acquaintances reproach me for my HIV infection.

Friends and acquaintances think badly of me because of my HIV infection.

Friends and acquaintances pity me excessively because of my HIV infection.

(Sex) partners reject me because of my HIV infection.

Because of my HIV infection, friends and acquaintances avoid me.

Because of my HIV infection, family members avoid me.

I am denied medical help because of my HIV infection, or I am treated unfairly when visiting a doctor, a hospital or other places of the health system.
